# COVID-19, conflict, and non-communicable diseases among refugees

**DOI:** 10.1016/j.eclinm.2021.100813

**Published:** 2021-03-30

**Authors:** Amani Al-Oraibi, Laura B. Nellums, Kaushik Chattopadhyay

**Affiliations:** a Division of Epidemiology & Public Health, University of Nottingham, Nottingham City Hospital, Hucknall Road, Nottingham NG5 1PB, United Kingdom; b The Nottingham Centre for Evidence-Based Healthcare: A JBI Centre of Excellence, Nottingham, United Kingdom

Non-communicable diseases (NCDs) are highly prevalent among refugees [1]. This is alarming, considering that many NCDs are key risk factors for severe COVID-19 infection, intersecting with migration-specific factors putting refugees at increased risk of infection and poor outcomes [2]. Prior to, during, and following migration, these populations are exposed to socioeconomic stressors including poor and overcrowded living conditions, limited access to water, sanitation, and hygiene (WASH) services, and linguistic, cultural, and legal barriers to accessing timely and appropriate care, negatively impacting on NCD development and control [2,3].

Despite the inclusion of NCDs in the Sphere Handbook, and efforts to integrate NCD management and prevention in the programming systems of humanitarian actors, the necessary attention to NCDs among refugees and the long-term commitment required to tackle them has still not been achieved [4]. The COVID-19 pandemic has added one more hardship to the care of NCDs by increasing the burden on overstretched health systems, reducing capacity to address NCDs, and disproportionately affecting those with previous health conditions, particularly in low- and middle-income countries, where the majority of refugees reside [5]. Over 65% of such countries worldwide have not reported including NCD services in their response to COVID-19 [5]. The prolonged impact this has had on management of NCDs has exacerbated their health and economic burden, and made it more vital than ever to proactively tackle this syndemic [6].

Jordan, for example, has the second highest population per capita of refugees worldwide, with over 750,000 refugees of around 60 different nationalities [7]. The vast majority are Syrian refugees, approximately 660,000 of whom have been registered in Jordan since the beginning of the civil war in 2011 [7]. NCDs have been identified as the most common health need among Syrian refugees in Jordan [8]. However, the large influx of refugees, and now the COVID-19 pandemic, have increased pressures on the health system, exceeding its ability to provide adequate care. This is cause for major concern, and further amplifies the already growing prevalence of NCDs in Jordan.

A cross-sectoral ‘health in all policies’ approach [9] is needed to create effective context-specific interventions and self-management plans for NCDs in the context of COVID-19. Governments, stakeholders, and policymakers involved in the provision of social and health services for refugees must partner together to implement rigorous management plans necessary to address and control NCDs among this population in these unprecedented circumstances. Additionally, international and non-governmental organisations should consider seeking diversified funding, aiding in prioritisation for equitable policy change, and preventative interventions for NCDs.

The effective prevention and management of NCDs will require a holistic approach which also addresses the protracted environmental and social determinants of disease in refugee communities, not only treatment. Interventions must target excluded groups based on linguistic, cultural, or legal factors, and both primary and secondary prevention initiatives are needed to address population-level determinants, as well as to facilitate detection, treatment, and self-management at the patient level. Effective interventions may include digital health tools (facilitating engagement with services, accessibility of records including self-held records, and follow-up and remote patient monitoring), outreach (e.g., mobile clinics, patient and public involvement), community-based interventions (e.g. collaborations with community organisations, religious centres), and capacity-building and awareness raising among both healthcare providers and refugees. In particular, clear, consistent, and accessible public health messaging should be provided to patients with NCDs about their increased risk of severe COVID-19 infection to increase their awareness and to support self-management of NCDS [10].

From our own experience of working with refugees in Jordan and other humanitarian contexts, we advocate for giving attention to their long-term – not only acute – health needs, and engaging these communities in future intervention plans to improve the quality of healthcare provision. We also call for research to address NCD management in humanitarian settings, incorporating the voices of refugees on the availability, feasibility, accessibility, and acceptability of NCD health services to optimise health system responses and the quality of the services these populations receive.

This pandemic underscores why addressing NCDs is crucial for health, development, and global security, and why forcibly displaced populations - who experience barriers to care, inequities in the social determinants of health- are disproportionately affected [3,5,9]. Humanitarian and receiving-country actors have a critical opportunity to harness the momentum generated during the pandemic to improve the provision of comprehensive NCD services to refugees, aligning with the Global Action Plan for the prevention and control of NCDs, and the 2030 Agenda for Sustainable Development Goal that committed to leaving no one behind.

## Declaration of Competing Interest

We declare no competing interests.
